# Similarity assessment by multivariate statistics method based on the distance between biosimilar and originator

**DOI:** 10.1186/s40643-021-00378-2

**Published:** 2021-03-29

**Authors:** Jian Xu, Zhihui Shao, Xiaoxiong Han, Yingfeng Huang, Xun Zou, Yaling Shen

**Affiliations:** 1 State Key Laboratory of Bioreactor Engineering, Shanghai Collaborative Innovation Center for Biomanufacturing Technology, East China University of Science and Technology, 130 Mei Long Road, Shanghai, 200237 China; 2 CAS Key Laboratory of Synthetic Biology, CAS Center for Excellence in Molecular Plant Sciences, Institute of Plant Physiology and Ecology, Chinese Academy of Sciences, Shanghai, China; 3 Process Development Department, Dragon Sail Pharmaceutical, Shanghai, China; 4 R&D Laboratories, Dragonboat Biopharmaceutical, Shanghai, China; 5 Shanghai Sanjin Bioscience and Technology, Shanghai, China

**Keywords:** Biosimilar, Cluster analysis, Glycosylation pattern, Multivariate statistics method, Similarity

## Abstract

The development of biosimilar products or follow-on biologics has been flourishing in recent years because of their lower price than the originators. In this study, a multivariate data analysis method based on JMP software was proposed to assess the glycosylation pattern similarity of antibody candidates from different conditions in optimization experiments with a reference. A specific distance was generated by this method and indicated the glycoform similarity between the biosimilar and the reference. This method can be applied to analyze the similarity of other physicochemical and functional characteristics between follow-on biologics and originators. Then, the design of experimental methods can be realized to optimize the conditions of cell culture to attain similar antibody candidates. A higher concentration of GlcNAc added to the basal media made the glycan of the antibody more similar to the glycan of the reference in this study.

**Supplementary Information:**

The online version contains supplementary material available at 10.1186/s40643-021-00378-2.

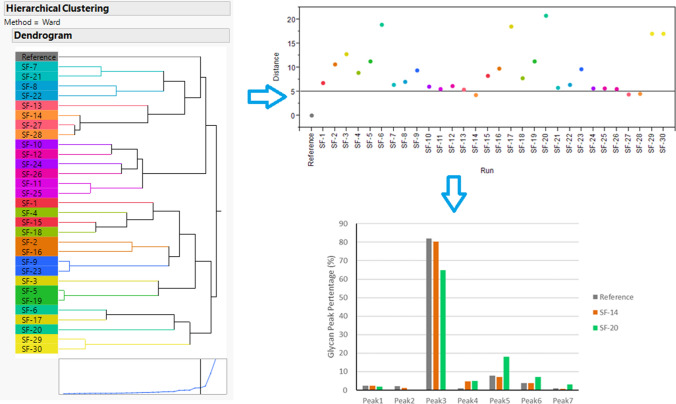

## Introduction

During the past two decades, biologic products (also termed “biopharmaceuticals”) have been developed intensively by companies for treating cancer and autoimmune diseases such as rheumatoid arthritis. Six of the top pharmaceutical product sales in 2019 were biologic products (Table S1, Additional file 1). Therefore, there has been an increasing trend toward the development of biosimilars, considering market interest and improved access to biologics. Biosimilar products can offer lower prices to optimize efficiencies across healthcare systems. However, biopharmaceuticals typically involve the expression of the gene using living cells followed by purification and formulation to acquire stable drug products. They are large, complex and heterogeneous compared to small molecule products. It is impossible to manufacture identical copies of biologic products (WHO 2016).

To define biosimilars, different regulatory bodies have different criteria. EMA defines it as a biotherapeutic product that is similar in terms of quality, safety, and efficacy to an already licensed reference product (reference medicinal product) in the EMA (EMA 2015). FDA defines it as a biological product that is highly similar to a US-licensed reference product notwithstanding minor differences in clinically inactive components and for which there are no clinically meaningful differences between the biological product and the reference product in terms of safety, purity, and potency of the product (FDA 2015). WHO defines it as a biotherapeutic product that is similar in terms of quality, safety, and efficacy to an already licensed reference product (WHO 2016). In broad terms, a biosimilar is highly similar to a reference product in terms of structure and function. High similarities of physicochemical and functional characteristics are the main aim in the preclinical development phase.

The structural and functional elements of therapeutic antibodies include the primary structure, purity, charge heterogeneity, glycosylation and other posttranslational modifications, as well as target and receptor binding activity and bioactivity features (Kirchhoff et al. 2017). A variety of analytic techniques have been developed to demonstrate these elements. However, many analytic results are multivariate data, such as charge heterogeneity, glycosylation and size heterogeneity. It is difficult to assess the similarity between biosimilars and originators (or references) based on these multivariate outcomes.

Identification of the glycosylation pattern is a key consideration during the development of monoclonal antibody (mAb) biosimilars, since the glycan chains in the Fc region can substantially alter protein activity and the PK profile and, in some cases, antigenicity (Kirchhoff et al. 2017). Because many cell culture conditions, such as nutrient availability, pH, dissolved oxygen (DO), ammonia, cell viability, growth phase, and temperature, will affect glycosylation, the culture process parameters should be well monitored and controlled during the manufacturing phase (Patrick et al. 2009). Chemical supplements such as metal ions and substrates for glycan chain synthesis have been described in the literature as effective glycosylation modulators during upstream process development (Crowell et al. 2007; Gramer et al. 2011). To find the optimal additive amount, the design of the experiment (DOE) is used intensively during the screening experiments. However, a specific parameter indicating similarity should be defined as the response before modeling and ANOVA. An effective method that can output a specific parameter to assess the similarity of glycan profiles is needed.

In this work, a multivariate data analysis method was first applied to generate a specific index which can represent the similarity of glycan profiles from different antibody candidates. By this method, similar antibodies were clustered by glycan distribution, and the most similar antibody was easily identified. The effect of supplements could be quantified statistically.

## Materials and methods

### Cell line and reagents

The cell line expressing recombinant IgG1 was derived from CHO DG44. The basal medium was CD DF1 (Shanghai BasalMedia Technologies Co., LTD., Shanghai, China) supplemented with 6 mM glutamine (Sigma, Shanghai, China). Feed media was Efficient Feed™ C + (Thermo Fisher, Shanghai, China), and additives were added as indicated in the design. The additives N-acetyl-D-glucosamine (GlcNAc) and MnCl_2_ were purchased from Sigma–Aldrich Shanghai Trading Co., Ltd. (Shanghai, China).

### Cell culture conditions and process design

The cells were cultured in 125 ml shaker flasks (Corning, New York, USA) with vented caps at 5% CO_2_, 70% humidity and 37 °C (shift to 35 °C from day 4 to harvest). The pH of the basal media was 7.0–7.2. The volume of the initial culture was 30 mL with a shaking rate of 120 rpm and a rotated diameter of 50 mm. The inoculum density was adjusted to 1 million cells/ml. GlcNAc and MnCl_2_ were supplemented into the culture in two ways: as an addition to the feed media or as an addition separately to the basal media on day 4. The supplemented amount was designed, as indicated in Table 1. The first 14 runs and second 14 runs were duplicated, and the last 2 runs were control runs without supplementation. The feed started on day 3, and the feed volume was calculated by Eq. (1):

1 $${V}_{\mathrm{feed}}={V}_{\mathrm{current}}\times {Q}_{\mathrm{feed}}\times \frac{\left({\mathrm{VCD}}_{1}+{\mathrm{VCD}}_{2}\right)}{2\times {\mathrm{Glc}}_{\mathrm{feed}}\times 1000}$$

where *V*_feed_ (mL) is the feed volume of the current day; *V*_current_ (mL) is the culture volume before feeding; *Q*_feed_ (g/10^9^ cells) is the specific glucose consumption rate in one day, which is 75 in this study; VCD_1_ (10^6^ cells/mL) is the viable cell density of the current day; VCD_2_ (10^6^ cells/mL) is the predicted viable cell density of the next day; and Glc_feed_ (g/L) is the glucose concentration in the feed media. The predicted viable cell density is double the current viable cell density in the exponential growth phase and equal to the current viable cell density in the steady growth phase.

**Table 1:** Design of the experiment for investigating the glycan profile adjustment in response to supplement addition

Run	GlcNAc (mM)^a^	MnCl2 (μM)^a^	Add in Basal or Feed Media^a^	Distance^b^
SF-1	120	30	Feed	6.71
SF-2	80	30	Feed	10.62
SF-3	40	30	Feed	12.75
SF-4	120	15	Feed	8.94
SF-5	80	15	Feed	11.27
SF-6	40	15	Feed	18.93
SF-7	120	0	Feed	6.44
SF-8	80	0	Feed	6.96
SF-9	40	0	Feed	9.42
SF-10	20	0	Basal	6.06
SF-11	10	0	Basal	5.45
SF-12	5	0	Basal	6.10
SF-13	10	30	Basal	5.42
SF-14	10	15	Basal	4.25
SF-15	120	30	Feed	8.22
SF-16	80	30	Feed	9.74
SF-17	40	30	Feed	18.50
SF-18	120	15	Feed	7.78
SF-19	80	15	Feed	11.20
SF-20	40	15	Feed	20.79
SF-21	120	0	Feed	5.80
SF-22	80	0	Feed	6.40
SF-23	40	0	Feed	9.65
SF-24	20	0	Basal	5.62
SF-25	10	0	Basal	5.64
SF-26	5	0	Basal	5.48
SF-27	10	30	Basal	4.44
SF-28	10	15	Basal	4.51
SF-29	0	0	N/A	16.97
SF-30	0	0	N/A	16.95

SF-1–SF-14 and SF-15–SF-28 were duplicate runs, while SF-29 and SF-30 were controls

^a^Three factors (GlcNAc additive concentration, MnCl_2_ additive concentration and the addition mode) were investigated in this experiment. Three levels of GlcNAc concentration in the basal media or feed media were tested. Three MnCl_2_ concentrations were tested in the basal or feed media. The experiment was conducted in a shaker flask culture system.

^b^The column is the distance value from cluster analysis, which are the response of the experiment

Sampling was conducted every day from day 3. The residual glucose was analyzed by a glucose test kit (Beihai, China). Additive glucose was calculated by Eq. (2) and added to maintain the concentration of glucose at 3 g/L when the viable cell density was less than 1.0 × 10^7^ cells/mL and shifted to 4 g/L when the viable cell density was higher than 1.0 × 10^7^ cells/mL:

2 $${V}_{\mathrm{Glc}}={\left[\left({\mathrm{Glc}}_{\mathrm{target}}-{\mathrm{Glc}}_{\mathrm{test}}\right)\times {V}_{\mathrm{current}}+\left({\mathrm{Glc}}_{\mathrm{target}}-{\mathrm{Glc}}_{\begin{array}{c}feed\\ \end{array}}\right)\times {V}_{\mathrm{feed}}\right]/{\mathrm{Glc}}_{\mathrm{solution}}}$$

where *V*_Glc_ (mL) is the additive volume of glucose solution; *V*_current_ (mL) is the culture volume before feeding; *V*_feed_ is the volume of feed media; Glc_feed_ (g/L) is the glucose concentration in the feed media; Glc_test_ (g/L) is the glucose concentration in the culture; Glc_target_ (g/L) is the target glucose concentration after feeding; and the Glc_solution_ (g/L) is the concentration of the glucose solution.

After 11 days of culture, the culture was harvested by centrifugation to remove the cell debris and the target antibody was purified by Protein A resin.

### Glycan analysis

#### Enzymolysis of glycan chains from the antibody

First, the buffer of the antibody sample was replaced with 50 mmol/L NH_4_HCO_3_ (pH 8.0). Then, 500 μg antibody was hydrolyzed with 2000 U PNGase F (New England Biolabs, Beijing, China) at 37 °C for 24 h. Precooled ethanol was added to a final concentration of 75% (v/v). The mixture was blended and allowed to stand for 0.5 h at − 20 °C. After centrifugation at 13,000 rpm for 15 min, the supernatant was vacuum dried.

### Fluorescence labeling of glycan chains

5 mg 8-aminopyrene-1,3,6-trisulfonic acid trisodium salt (APTS, AB Sciex Pte. Ltd., Framingham, USA) were added into 0.5 mL aqueous solution containing 15% acetic acid and vortexed it. Then, 15 μl APTS solution and 5 μL tetrahydrofuran solution containing 1 mol/L sodium cyanoborohydride were added to the vacuum-dried glycan chains. After fluorescent labeling at 55 °C for 2 h, 400 μl ultrapure water was added and the product was analyzed by a PA800 Plus capillary electrophoresis apparatus (AB Sciex Pte. Ltd., Framingham, USA).

### Analysis by a capillary electrophoresis apparatus

A Beckman N-Cho-coated capillary with a total length of 60.5 cm, effective length of 50 cm and inner diameter of 50 μm, and the electrophoresis buffer were purchased from Beckman Coulter Life Sciences (Indianapolis, USA). The capillary temperature was 20 °C. The sample was injected at 2.0 psi for 10 s. Then, the sample was separated at 30 kV for 20 min. Fluorescence detection was implemented at excitation and emission wavelengths of 488 and 520 nm, respectively.

### Multivariate statistics

The peak area percentages from the glycan analysis are used for assigning the similarity in this case. Seven peaks were identified and coded by numbers for each isoform. In this way, all of the data were normalized for each sample. A matrix of the data was built, and hierarchical cluster analysis was performed with JMP software (SAS Institute Inc.). A dendrogram could be generated and the distance matrix could be saved to another data table. The distance can refer the measure of virtual distance between biosimilar to originator. Distance can also be denoted similarity. To analyze the effect of the 3 factors on glycan similarity, the distance was set as the response value and then fit model was run in JMP.

## Results

### Glycan analysis

**Fig. 1:**
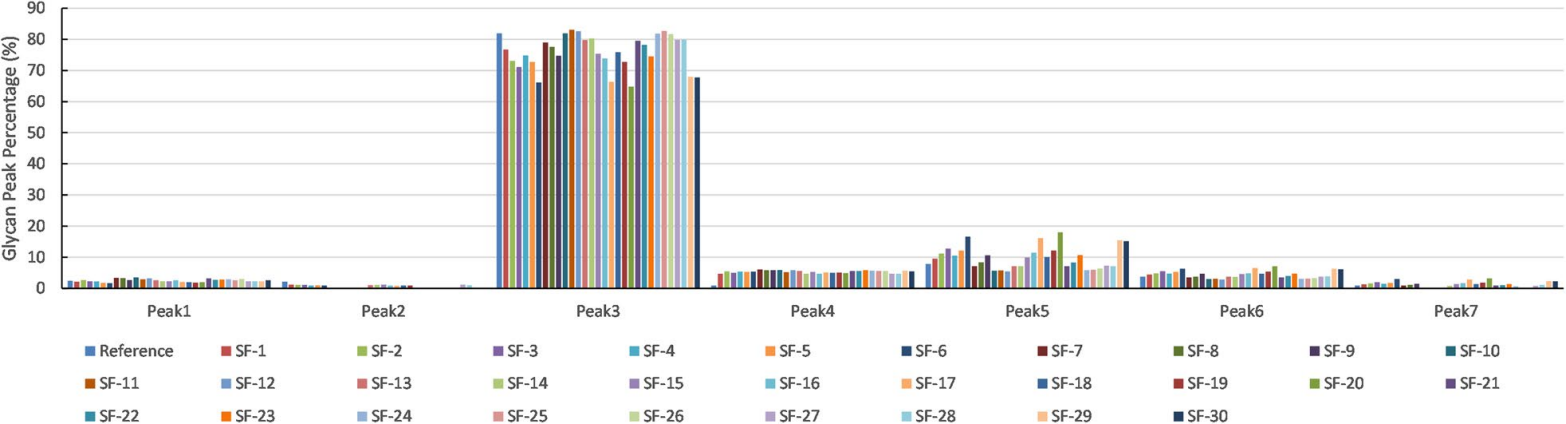
Glycan peak distribution of 30 samples from the shaker flask culture experiment. Each peak percentage denotes one glycoform from the antibody. Peak 3 is the most abundance glycan an peak 5 is the second portion. The reference glycan distribution is the first column in each peak

The cell growth data and residual glucose concentration variation are shown in the Additional file 1. The cells grew well, and glucose was not depleted in all culture runs. The culture was harvested on day 11, and the supernatant was collected by centrifugation. After purification and analysis as described in the methods, the glycan peak distributions of 30 samples from different shaker flask cultures are shown in Fig. 1. All of the samples’ glycans showed similar patterns, with peak 3 being the highest portion; however, the percentages of each peak were different. At a glance, supplementation with GlcNAc and MnCl_2_ should affect the glycan distribution of antibodies. However, it is difficult to recognize the effect of the supplements, and the effect cannot be tested by a statistical method, because a specific number to represent the similarity is lacking.

### Cluster analysis

**Fig. 2:**
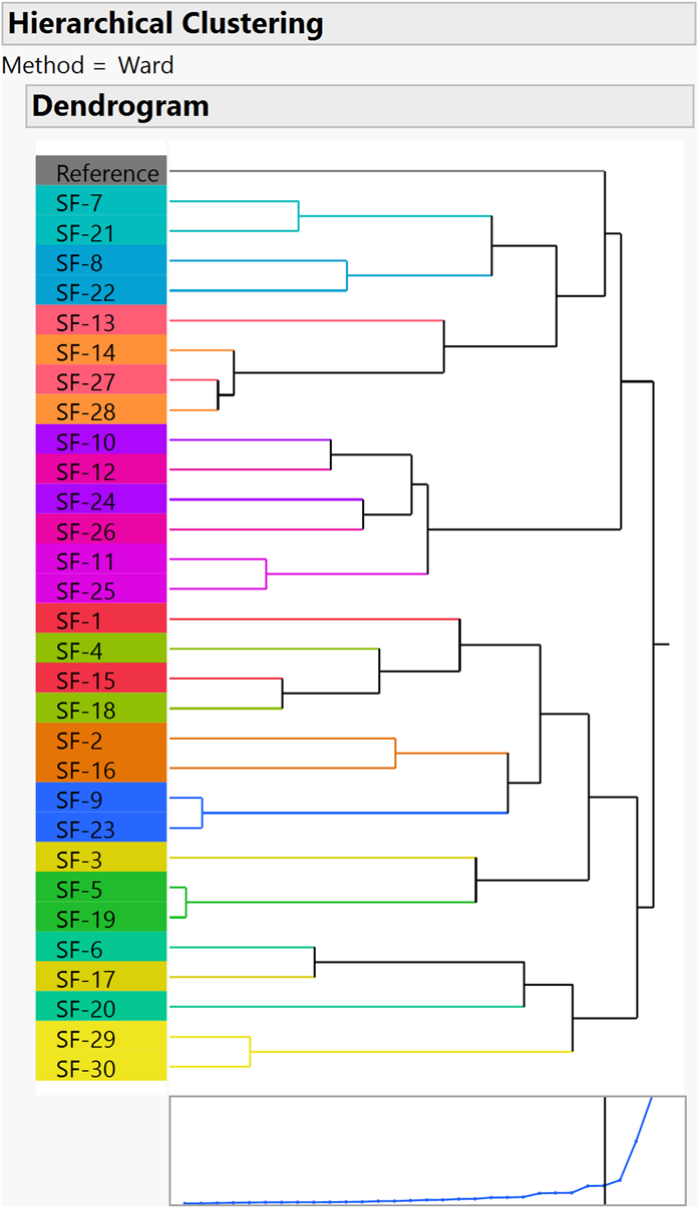
Hierarchical clustering tree analyzed by JMP software. The duplicated runs are highlighted with the same sample color. Similar glycan pattern samples are clustered into one trunk. The samples share the same trunk with the reference, which means that they are similar to the reference

A 31 × 8 matrix with a 7 peak area percentage of the reference and each sample was generated. By performing the cluster analysis tool in JMP as described in the Methods, a hierarchical clustering tree is shown in Fig. 2. The single samples are the leaves, and the similar samples are clustered on one branch. We set the duplicate runs to the same color, so it can be seen that almost all duplicate samples are in the third or fourth branch from the main trunk. It is very clear that SF-7, 8, 13, 14, 21, 22, 27, and 28 are located in the same third branch as the reference, which illustrates that the glycan distribution of these samples is very close to that of the reference.

### Distance

**Fig. 3:**
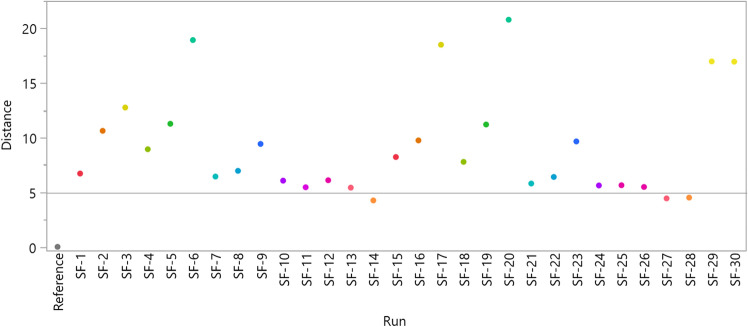
Euclidean distance of 30 antibody candidates to the reference. The dot color is the same as the color in the hierarchical clustering tree for the same samples. A shorter distance means more similar to the reference

**Fig. 4:**
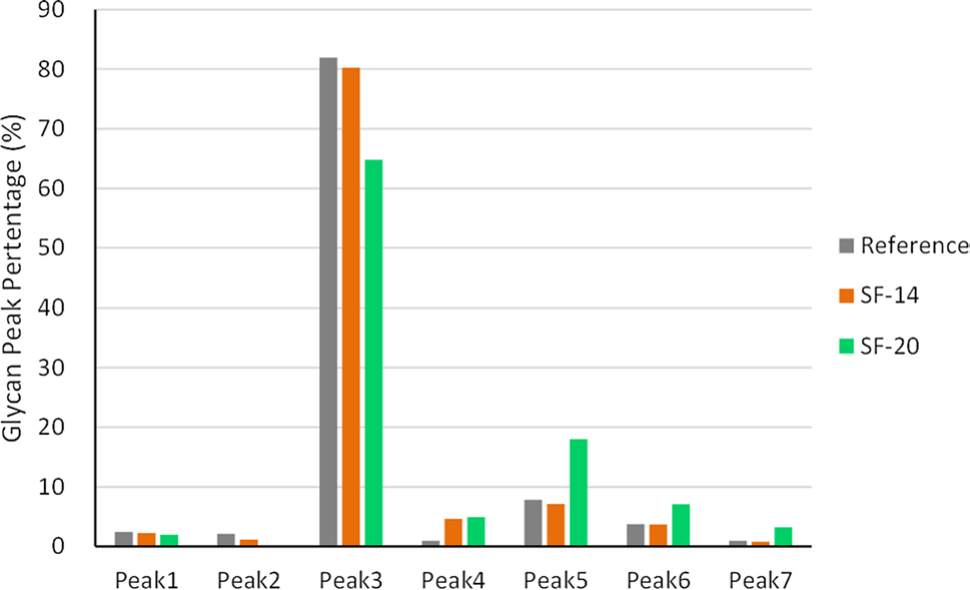
Glycan peak distribution similarity comparison of SF-14, SF-20 and the reference. The distance of SF-14 is the shortest to the reference, while the distance of SF-20 is the longest to the reference. The peaks percentage of SF-14 is similar to the peaks percentage of reference

The distance shows the similarity between the samples and the reference. They can be generated by saving the distance matrix option in menu. The distances are plotted to run the label, as shown in Fig. 3. Here, it is very clear that the antibodies from SF-14 and 28 are the nearest to the reference antibody in terms of glycan distribution, while SF-6 and 20 are the farthest. To confirm the real peak distribution similarity, only SF-14, SF-20 and the reference are shown in Fig. 4. SF-14 is similar to the reference, and the difference between SF-20 and the reference is significant.

### Effect Significance Analysis

Use the “Fit model” in JMP, we set the Distance as the response and put “GlcNAc”, “MnCl_2_” and “adding them in basal or feed media” in the model effect window and then ran the fit program. The ANOVA results are shown in Table 2. In the effect test, the *P* value of GlcNAc additive concentration and addition to basal or feed media were < 0.0001, which means that these 2 factors were significant, while the MnCl_2_ additive concentration was 0.1083, which was justified as not significant. A higher concentration of GlcNAc added to the basal media made the glycan of the antibody more similar to the glycan of the reference. In this way, the significance of the factors can be identified statistically, and then the optimal condition can be quickly determined.

**Table 2:** ANOVA results of three factors affecting glycan similarity using the distance value as the response.

Source	Nparm	DF	Sum of Squares	F Ratio	P
GlcNAc	1	1	173.1	27.2	<0.0001*
MnCl_2_	1	1	17.6	2.8	0.1083
Add in Basal or Feed Media	2	2	474.2	37.3	<0.0001*

*Significant when *P* is less than 0.05

## Discussion

In developing a biosimilar drug, a stepwise approach is needed beginning with chemistry manufacturing controls (CMC) and bioanalytical characterization (Burchiel et al. 2019). In the CMC development phase, because the structure of antibodies is very complex, the quality attributes will be characterized by multiple analysis methods, such as glycan isoforms, capillary isoelectric focusing (cIEF), cation-exchange chromatography (CEX) and peptide maps. We know that these methods output multivariance results rather than single data. Clustering is a ubiquitous data analysis tool to divide complex data into groups of similar items (Andreas et al. 2019). Therefore, it can be utilized to reveal the similarity of these multivariance test results effectively. Kang and Chow (2013) proposed a three-arm parallel design to assess biosimilarity between a biosimilar product and an innovator biological product based on the relative distance of means observed from the test and reference products. In the proposed design, if the relative distance is less than a prespecified margin, they claim that the two products are biosimilar. This method’s merit is to provide a specific standard to access similarity, but it is relatively complex and inexecutable compared to the method we proposed. Beyond this method, we have not found other strategies to assess biosimilar quality by a statistical pathway in the published literature.

The clustering program in JMP can output a hierarchical tree, making the cluster results visualizable and easier to find a similar group to a reference. This can be utilized to control the batch quality in the manufacturing phase. If the third branch is set as a similar margin, the leaves of different batch data can be judged as qualified batches. Once a batch’s quality is clustered to another third branch, deviation investigation can be triggered to recall or destroy the batch based on the risk assessment. The distance between samples and the reference can be used as the response value in the optimization experiment. In this way, a quality-by-design (QbD) strategy is feasible to characterize the process effect and to optimize or define the operation space of the critical process parameter (CPP) by DOE. In the future, many batches will be clustered, and a distance can also be set as a margin to select the qualified batch.

GlcNAc is one of the main monosaccharides in conserved N-linked glycan structure. In the medial Golgi, the N-acetylglucosaminyltransferase–I (GnTI) enzyme mediates the transfer of GlcNAc from UDPGlcNAc to the O-2 position of the terminal mannose residue in the α1 → 3 branch of the Man5GlcNAc2 oligosaccharide (Liming 2015). GlcNAc supplement will increase the substrate of glycan synthesis and change the percentage of various glycans. In this study, the additional glycans increased the percentage of peak 3 and decreased the percentage of peak 5, 6 and 7 significantly. Crowell et al. (2007) reported that manganese is the cofactor of galactosyltransferse and increased the galactosylation. For the antibody we studied, effect of MnCl_2_ is not significant in our tested range but it may help GlcNAc to tune galacosylation to be more similar to reference.

## Conclusion

In this paper, a multivariate statistics method is proposed to assess the similarity of antibodies to references from different conditions in optimization experiments. The multivariance quality results can be grouped by this method, and a specific distance can be generated. The distance value indicates the similarity between the biosimilar and the reference and the DOE method can be realized to evaluate the effects of factors and to optimize the culture conditions. In the case study, the highest similar glycoform was easily identified. GlcNAc supplemental and adding mode were significant factors and their impact were clear. The optimal conditions to attain higher similar antibody were 10 mM GlcNAc and 15 μM MnCl_2_ supplemented into basal medium.

### Supplementary Information

**Additional file 1:** **Table S1.** The top 10 pharmaceutical products sold in 2019. **Fig. S1.** Viable cell density variation of 30 shaker flask runs by the culture time. **Fig. S2** Viability variation of 30 shake flask runs by the culture time. **Fig. S3** Residual glucose variation of 30 shake flask runs by the culture time.

## Data Availability

Not applicable.

## Abbreviations

WHO: World Health Organization
EMA: European Medicines Agency
FDA: U.S. Food and drug administration
mAb: Mono-clonal antibody
PK: Pharmacokinetics
DO: Dissolved oxygen
DOE: Design of experiment
ANOVA: Analysis of Variance
GlcNAc: N-Acetyl-D-glucosamine
CMC: Chemical, manufacture and control
cIEF: Capillary isoelectric focusing
CEX: Cation-exchange chromatography
QbD: Quality by design
CPP: Critical process parameter
