# Supplementary material for: Similarity assessment by multivariate statistics method based on the distance between biosimilar and originator
Source: Bioresour Bioprocess. 2021 Mar 29;8(1):24. doi: 10.1186/s40643-021-00378-2 (PMC10992075; doi:10.1186/s40643-021-00378-2)
Supplement: Supplementary file 1 — Additional file 1: Table S1. The top 10 pharmaceutical products sold in 2019. Fig. S1. Viable cell density variation of 30 shaker flask runs by the culture time. Fig. S2 Viability variation of 30 shake flask runs by the culture time. Fig. S3 Residual glucose variation of 30 shake flask runs by the culture time. [file 40643_2021_378_MOESM1_ESM.docx]

**Table 1** The top 10 pharmaceutical products sold in 2019*.

| Rank | Medicine | Sales (Billion $) | Company | Type |
| --- | --- | --- | --- | --- |
| 1 | Humira | 19.17 | Abbvie | Antibody |
| 2 | Eliquis | 12.15 | BMS/Pfizer | Small Moledular |
| 3 | Keytruda | 11.08 | MSD | Antibody |
| 4 | Revlimid | 7.171 | Celgene | Small Moledular |
| 5 | Imbruvica | 8.09 | J&J/Abbvie | Small Moledular |
| 6 | Opdivo | 8.00 | BMS | Antibody |
| 7 | Eylea | 7.44 | Bayer/Regeneron | Fusion Protein |
| 8 | Avastin | 7.12 | Roche | Antibody |
| 9 | Xarelto | 6.93 | Bayer | Small Moledular |
| 10 | Enbrel | 6.92 | Amgen/pfizer | Fusion Protein |

**Note:** * Data from the annual statement of the company.

**Fig.1** Viable cell density variation of 30 shaker flask runs by the culture time

**Fig. 2** Viability variation of 30 shake flask runs by the culture time.

**Fig. 3** Residual glucose variation of 30 shake flask runs by the culture time.
